# Health-Related Lifestyles among University Students: Focusing on Eating Habits and Physical Activity

**DOI:** 10.3390/ijerph21050626

**Published:** 2024-05-15

**Authors:** Elena Lonati, Emanuela Cazzaniga, Roberta Adorni, Francesco Zanatta, Michael Belingheri, Matteo Colleoni, Michele Augusto Riva, Patrizia Steca, Paola Palestini

**Affiliations:** 1 School of Medicine and Surgery, University of Milano-Bicocca, Via Cadore, 48, 20900 Monza, Italy; emanuela.cazzaniga@unimib.it (E.C.); michael.belingheri@unimib.it (M.B.); michele.riva@unimib.it (M.A.R.); paola.palestini@unimib.it (P.P.); 2 Department of Psychology, University of Milano-Bicocca, Piazza dell’Ateneo Nuovo, 20126 Milan, Italy; f.zanatta@campus.unimib.it (F.Z.); patrizia.steca@unimib.it (P.S.); 3 Department of Sociology and Social Research, University of Milano-Bicocca, Piazza dell’Ateneo Nuovo, 20126 Milan, Italy; matteo.colleoni@unimib.it; 4 BASE Bicocca Sustainability Committee, University of Milano-Bicocca, Piazza dell’Ateneo Nuovo, 20126 Milan, Italy

**Keywords:** lifestyle, diet, eating habits, physical activity, age, gender, university students, protein intake

## Abstract

The transition to higher education at University is a critical moment for young adults to acquire unhealthy habits regarding physical activity (PA) and adherence to a healthy diet. Negative behaviors might be maintained in the years to come with a major risk of suffering from a Non-Communicable Disease. This study aims to determine the relationship between diet and PA in the student community of University of Milano-Bicocca. Students between 18 and 30 years old completed an online survey (6949 students). Two analyses of covariance (ANCOVA), chi-square tests of independence, and a binomial logistic regression were performed to examine the relationship between adequacy of food consumption and PA, in association also with sociodemographic characteristics. Data show a strong correlation between behaviors analyzed, with a proportional positive association between PA and healthy diet. Nevertheless, a third of the sample students incur in incorrect habits for both diet and PA. Further, students performing intensive PA have the healthiest food consumption in general but the worst red meat and pork intake. Accordingly, men practice more PA but have a less adequate diet, exactly contrary to women. In conclusion, policies promoting consciousness of well-being would transform Universities into healthy hubs for virtuous habits.

## 1. Introduction

Nowadays, about 74% of all deaths worldwide are due to Non-Communicable Diseases (NCDs), including heart disease, stroke, cancer, diabetes and chronic lung disease. The World Health Organization (WHO) aimed to improve international action on surveillance, prevention and control of NCDs according to the United Nations 2030 Agenda for Sustainable Development. Indeed, in 2019, it was necessary to extend the WHO Global action plan for the prevention and control of NCDs 2013–2020 [1] in order to fight against them. A healthy lifestyle still represents the main strategy to counteract the four major risk factors shared by NCDs: tobacco smoking, physical inactivity, excessive alcohol use and unhealthy diets [2,3]. Since the combination of two or more of these risk factors exacerbates the impact of a single one [4], the worldwide community needs to proceed with a “multiple health behavior change” approach that also takes into account social, economic and environmental factors [5]. Although interventions at each step of life are important to change unhealthy habits, it is fundamental to investment in young people’s health to protect educational programs regarding sustainable food choices made in childhood. The acquisition of detrimental habits during adolescence and youth often results in health problems in adulthood. In this context, transition to higher education in university is a critical moment [2,6,7]. During their University career, several students adopt unhealthy routines, reducing the time dedicated to physical activity [8] and adherence to a balanced diet. Indeed, they skip breakfast, eat snacks for brevity and increase sugar, fat and sodium intake, together with a decrease in fruit and vegetable consumption [9,10,11,12]. 

Currently, several studies have demonstrated that adherence to the Mediterranean diet is associated with lower risk of cardiovascular disease, metabolic syndrome and abdominal obesity [12,13], and that it is strictly correlated to physical activity (PA) engagement [12,14]. In 2020, Zeppa and colleagues also reported that high intensity exercise led to adopting healthy diet choices [15]. Accordingly, a correct nutrition plan for athletes is becoming an imperative to reach the prefixed objectives [16]. Food choices concerning what to eat before and after a PA effort determine the best performance and better recovery afterwards [17,18]. The 4Rs framework for nutritional strategies for post-exercise is key to performance in sport: rehydration; refuel—the consumption of carbohydrates; repair—post-exercise ingestion of high-quality protein; and Rest—pre-sleep nutrition [19]. People performing intensive PA are normally used to a Western diet based on meals containing animal-derived protein [20]. Given this, they may tend to adopt incorrect behaviors concerning red meat intake with the idea or arriving at the best form of protein supply.

Therefore, in a survey whose general purpose was to explore the entire lifestyle of the total academic community [21], this study focuses on two important aspects of healthy behavior in students: diet and physical activity. The main objective is to determine the relationship between the adherence to healthy diet and PA, also exploring choices in relation to sociodemographic characteristics. Moreover, a particular focus on intensive PA and red meat consumption, related also to gender, is carried out. Indeed, in recent research, Sares-Jäske and colleagues revealed that men more than women consider meat consumption very important in their diet, with this specific attitude common among younger adults [22]. 

## 2. Materials and Methods

### 2.1. Participants and Procedure

The study was conducted from May to June 2019 through an online survey using snowball sampling. All students and employees of the University of Milano-Bicocca, a prominent university in northern Italy, were eligible to participate. The online survey was created, piloted, and administered using the LimeSurvey platform and gathered data on sociodemographic indicators and lifestyles, including diet, physical activity, alcohol consumption, and cigarette smoking. The first page of the survey outlined the study’s objective and requested participants to provide their digital informed consent, indicating that they had read and accepted the privacy regulations. Participation was voluntary and anonymous, and participants could withdraw from the study without any consequences. 

A previous paper [21] offered a general overview of the results, including both students and employees, and considered the four aspects of lifestyle. This paper focused on university students between 18 and 30 years old and considered diet and physical activity. A total of 6949 students completed the survey. Their mean age was 22 years (SD = 2.30); 71% were women. Most participants in the sample were enrolled on a bachelor’s degree course (58.9%), 25.4% attended a master’s degree course, 15.1% attended a single-cycle degree course, and 0.6% were in a postgraduate master’s degree.

This research adhered to The Code of Ethics of the World Medical Association (Declaration of Helsinki) for experiments involving humans. The study was conducted as an internal company survey, and as such a formal collaboration agreement was signed with the Director of the University of Milano-Bicocca prior to data collection. This agreement attested to the study’s objectives and confirmed compliance with the privacy policy for data processing. Additionally, a positive legal opinion was provided by the General Director of the University. The study was also presented to and discussed with the Director and President of the University Committee of BASE (Bicocca Alimentazione Sostenibilità ed Economia), who expressed a favorable opinion.

### 2.2. Lifestyle Measures

#### 2.2.1. Diet

Study participants were asked to rate the frequency of their consumption of various food types using a 5-point Likert scale. The foods included bread, pasta, rice, cold cuts, white meats, red meats, dairy products, eggs, fish, fruit and vegetables, legumes, salty snacks, and sweets. Ratings ranged from “More than once a day” (1) to “Never” (5). They were also asked to indicate which fats they used most often for cooking and seasoning foods, choosing from olive oil, other vegetable fats, or butter/lard. Participants then reported on their level of attention to salt consumption using a 3-point Likert scale, with options ranging from “I have never paid attention” (1) to “I have always paid attention” (3). These survey questions (listed in the Supplementary Materials Tables S1–S3) were adapted from the National Institute of Statistics [23]. Regardless of how different eating behaviors were assessed, they were classified as adequate (score = 1) or non-adequate (score = 0), following international [24] and national [25] guidelines. For statistical analysis, we calculated a diet adequacy index by summing the scores for specific foods, similar to the MedDietScore scale [26], resulting in a variable diet synthesis. Higher scores indicate greater diet adequacy. Finally, we classified participants’ diet as non-adequate (score between 1 and 9), sufficiently adequate (score between 10 and 15), or fully adequate (score ≥ 16) to provide a brief overview of their eating habits.

#### 2.2.2. Physical Activity

Participants were asked to report on their physical activity levels using a 4-point Likert scale that ranged from “Never” (1) to “Five or more days a week” (4) (see Supplementary Materials Table S4). This activity scale was based on a survey from the National Institute of Health [27] and included three types of physical activity: light, moderate, and intense. Each response was assigned a score between zero and four, taking into account both the frequency and intensity of the activity, in accordance with international [28] and national [27] guidelines.

To create a physical activity adequacy index for statistical analyses, we added the scores obtained for each response, a similar method to how we calculated the diet scores. This provided a synthesis of the physical activity variable, with higher scores indicating greater adequacy of physical activity. To briefly describe the participants’ physical activity behavior, we classified their scores as non-adequate (score between 0 and 1), adequate (score between 2 and 5), and intensive (score ≥ 6).

### 2.3. Data Analysis

We calculated descriptive statistics for the sample’s sociodemographic and lifestyle characteristics. Our report includes mean and standard deviation (SD) for continuous variables and percentages for categorical variables. We performed a preliminary analysis on the two synthesis lifestyle variables to assess for normal distribution. This involved calculating mean, SD, and indices of skewness and kurtosis; West and collaborators (1995) [29] (pp. 56–75) recommend concern if skewness > |2| and kurtosis > |7|.

Two analyses of covariance (ANCOVA) were performed. The first considered the synthesis diet variable as the dependent variable. The independent variables included gender (2 levels: male and female), age (covariate), and adequacy of physical activity (3 levels: non-adequate, adequate, and intensive). The second ANCOVA considered the synthesis physical activity variable as the dependent variable. The independent variables included gender (2 levels: male and female) and age (covariate). Post-hoc Bonferroni tests were used to compare means across multiple groups. Assumption checks were performed by evaluating skewness and kurtosis to ensure the normal distribution of the variables and Levene’s test was used to assess the homogeneity of variances.

A series of chi-square tests of independence were performed to examine the relationship between the adequacy of food consumption (adequate vs. non-adequate) and physical activity (non-adequate, adequate, and intensive), as well as the adequacy of food consumption and gender.

A binomial logistic regression analysis was performed to investigate the relationship between the adequacy of beef consumption, physical activity, and gender. The adequacy of beef consumption (adequate vs. non-adequate) was treated as the dependent variable; physical activity (inadequate, adequate, and intensive) and gender (male vs. female) were included as categorical independent variables to evaluate their potential interaction.

For all the analyses performed, a *p*-value ≤ 0.05 was considered statistically significant. The data analyses were conducted using IBM SPSS Statistics for Windows, version 26.0 (IBM Corp., Armonk, NY, USA), and Jamovi (Version 2.2.5, The Jamovi project, 2021, retrieved from https://www.jamovi.org, accessed on 21 August 2023).

## 3. Results

### 3.1. Description of Diet and Physical Activity

Overall, most of the sample demonstrated an adequate consumption of the different food groups (Table 1). Exceptions were cured meats, beef, and sweets with too high consumption, and leafy vegetables and dried or canned legumes, with too low consumption (See Supplementary Materials Tables S1–S3).

Although the majority of participants exhibited a sufficiently healthy diet (68.8%) and performed adequate or intensive physical activity (59% and 7%, respectively), a significant proportion of students at the University of Milano-Bicocca demonstrated non-adequate behaviors in both areas (Table 2). Moreover, the percentage of students performing adequate physical activity was lower than those with a sufficiently healthy diet. For a detailed description of the individual behaviors, see Supplementary Materials Tables S4 and S5.

### 3.2. Association between Diet, Physical Activity, and Sociodemographic Characteristics

Descriptive statistics for all variables of interest are reported in Table 2. All variables were normally distributed.

The first ANCOVA was performed considering the synthesis diet variable as the dependent variable; sociodemographic characteristics and adequateness of physical activity were the independent variables. Assumption checks suggested that the group variances were homogeneous (Levene’s test = 0.55; *p* = 0.74). Results highlighted a significant effect of gender (F = 90.82; *p* < 0.001; η^2^p = 0.013). Women had a more adequate diet (Mean = 11.5; SE = 0.06) than men (Mean = 10.6; SE = 0.07). Older age was associated with a more adequate diet (F = 50.36; *p* < 0.001; η^2^p = 0.007; β = 0.083). Finally, a statistically significant effect of adequacy of physical activity emerged (F = 80.01; *p* < 0.001; η^2^p = 0.023). Participants performing intense physical activity had the most adequate diet (Mean = 11.6; SE = 0.11), followed by participants performing adequate physical activity (Mean = 11.1; SE = 0.04). Participants performing non-adequate physical activity had a less adequate diet (Mean = 10.35; SE = 0.06). Post-hoc comparisons showed that all contrasts were significant at the *p* < 0.001 level. The interaction between gender and adequateness of physical activity did not reach statistical significance (*p* = 0.077).

The second ANCOVA was performed considering the synthesis physical activity variable as the dependent variable and sociodemographic characteristics as the independent variables. Assumption checks suggested that the group variances were not homogeneous (Levene’s test = 82.9; *p* < 0.001). Therefore, the parameters’ estimation with robust standard errors was performed. Results highlighted a significant effect of gender (F = 356.5; *p* < 0.001; η^2^p = 0.049). Women performed less physical activity (Mean = 2.07; SE = 0.03) than men (Mean = 3.01; SE = 0.04). Older age was associated with less adequate physical activity (F = 18.4; *p* < 0.001; η^2^p = 0.003; β = −0.050).

### 3.3. Association between Specific Foods’ Consumption and Physical Activity

Generally, the greater the physical activity the students perform, the greater the diet adequacy. This evidence emerged as statistically significant for the consumption of fruit and vegetables, legumes, and sweets and in the use of seasoning and salt. Regarding animal proteins, this association was verified for the consumption of eggs and fish. In contrast, in the case of red meat and pork, less adequate consumption emerged in participants who practiced more physical activity (Table 3).

### 3.4. Association between Specific Foods’ Consumption and Gender

Generally, women had a more adequate diet than men. This evidence emerged as statistically significant for the consumption of cured meats, beef, pork, vegetables, fruit, salty snacks, and salt. In contrast, men consumed cereals, white meat, dairy products, eggs, and potatoes more adequately than women (Table 4).

### 3.5. Association between Beef Consumption, Physical Activity and Gender

**Figure 1:**
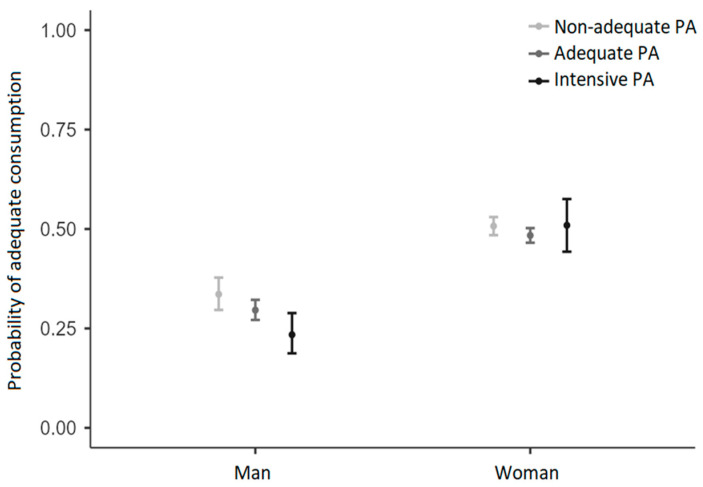
Distribution of the probability of beef consumption adequacy as a function of gender and physical activity (PA).

The binomial logistic regression results indicated that the full model was statistically significant, χ^2^ (5, N = 6947) = 242, *p* < 0.001, suggesting that it could distinguish between respondents who consumed an adequate amount of beef and those who did not. The model explained between 3.4% (Cox and Snell’s R2) and 4.6% (Nagelkerke’s R2) of the variance in beef consumption adequacy. As shown in Table 5, both gender (*p* < 0.001) and intensive physical activity (*p* < 0.005 for the contrast with insufficient PA; *p* < 0.05 for the contrast with adequate PA) were associated with beef consumption. The odds ratio indicated that women were two times more likely to report adequate consumption than men (OR = 2.035). Regarding physical activity, the odd ratios indicated that respondents who practiced intensive PA were one and a half times more likely to report non-adequacy than respondents who practiced insufficient PA (OR = 0.605) and adequate PA (OR = 0.727). The interaction between gender and physical activity suggested that non-adequacy was particularly evident in men who practiced intensive PA (see Table 5 and Figure 1).

## 4. Discussion

This study provided interesting results in the field of health-related lifestyles considering that analyzes in university student communities are relatively few, even more so among Italian Universities. One of the study’s strengths is its involvement of around 7000 students; therefore, the data obtained are representative of young people in the 18–30 age group.

Our data showed that, in 2019, in a pre-COVID19 period, a third of the sample engaged in incorrect behaviors for diet and physical activity (PA). In line with other studies, in our community, a trend was registered of an inadequate higher consumption of cured meats, beef and sweets versus a lower intake of vegetables and legumes [7,9]. Indeed, the transition to university life led to different habits and time management with respect to high school-life. In the teenage years, nutrition choices were influenced by the family contest and the daily plan was precisely marked with time dedicated to PA, whereas in the university years the increased autonomy, the small food budgets, the necessity of fast meals and the exposure to new social groups might determine important changes [30].

Nevertheless, a strong correlation between the behaviors analyzed has emerged from this survey. Those who do not engage in physical activity are also those who have the lower adherence to diet guidelines. This result agrees with a survey administered to Italian young people (ages 17–35) by La Fauci and colleagues in the same period [31]. Among those who practice PA, the greater the PA performed, the better the adequacy of the diet. Probably, common motivation such as body image and well-being are at the base of healthy choices. Further, the higher PA performers also pay attention to nutrition for their sportive performance. The training must be accompanied with an accurate plan of nutrient intake for the different daily meals, with particular carefulness to pre- and post-exercise moments in order to minimize muscle damage and facilitate greater acute and chronic training adaptations [2,32]. Accordingly, according to data reported in different studies [5,12,15,33,34], students performing intensive PA present a more adequate consumption of fresh fruit, vegetables, fish and eggs with respect to other groups. On the other hand, the same survey participants have the most inadequate consumption of red meat and pork, probably because they are interested in increasing muscle mass with elevated protein intake. Endurance and strength-power athletes often apply a strategy that foresees a combined carbohydrate + protein enriched food consumption in order to ameliorate their performance. Moreover, the intensive PA performers were the best legume consumers in the study. Indeed, many athletes in these categories have adopted legumes as protein sources in their diet [35,36]. Nevertheless, adequate legume intake in all groups is under 50% with respect to the guidelines. This evidence opens possibilities of a targeted intervention aimed to move their food choices toward protein of vegetal origin.

As happened in precedent studies [7,12,13,34], women represent the majority of the sample (70%), probably because females are more predisposed to survey response. Interestingly, sociodemographic characteristics (gender and age) have a significant impact on student choices about nutrition and PA. Even if women show more adherence to a healthy diet, men practice more physical exercise and sport activity. This evidence is probably related to the different motivations that drive women and men to PA. Variables related to the social environment, such as competition and social recognition, determine men’s approach to PA, while women are more careful about weight control [37]. This motivation would also explain why women pay more attention to correct food intake. In a preceding study carried out in an Australian university student community, several discrepancies were observed between men and women, revealing men’s worst behavior in “discretionary choices”, i.e., foods high in saturated fat, added salt and added sugars [38]. In several countries, men typically also eat more meat than women [34,39,40], while females are more likely to vegetarianism and veganism choices, in general and also among athletes [41]. Here we show that men with the worst behavior regarding meat consumption belong to the intensive PA performer group. As mentioned above, this behavior is probably driven by the desire to ameliorate performance in competition and muscle definition [42].

It is worth noting that older students are less engaged in PA. Keating and colleagues found through a meta-analysis approach that there were some indications of sedentary increase during last years of studying [43]. Probably, a student’s later university career leads to an intensification of workload and a major interest in academic scores, leaving PA habits in favor of academic performance. On the other hand, a positive association between older age and healthy diet adherence is revealed, suggesting that consciousness of dietary choices increases with age. Students become aware of the nutritive power of foods consumed and their importance in well-being at both physical and mental levels [7,44].

This picture given of the Milano-Bicocca student community might have changed in the last four years due to the impact of the COVID-19 pandemic on eating and PA habits in the population in general, Interesting papers have highlighted the short-term effects of lockdown and the following rebooting [45,46,47]. People introduced some favorable changes in food intake, such as an increase in raw vegetables and whole grains, that were maintained in the successive months after lockdown [48] Moreover, the Italian population’s eating choices changed, with a major adherence to the Mediterranean diet, especially in the 18–30 years range [49]. Concerning PA, during lockdown there was a strong change in habits: athletes, scholars and active people were constrained to reduce their training time due to government restrictions, while sedentary people were induced by home confinement to introduce some training into their daily schedule. However, during rebooting, people returned to practice different types of PA based on age and gender [47], and in the long-term post-COVID 19 vision there was a “return to normal” for athletes and scholars and for active people [48]. These considerations taken together lead us to speculate that the Milano-Bicocca student community might have improved its dietary habits, maintaining or even increasing time spent in PA. 

### Limitations

This study provides interesting and significant findings, in line with evidence collected by several recent studies carried out in different communities worldwide. However, it is a picture of a single University in the Milan area, thus it would be important to amplify the catchment area to several Italian universities with a focus on differences among regions, from north to south. Moreover, although the questionnaire on PA was made to obtain information regarding the number of days and types of physical activity performed by each individual in order to define minutes of moderate and intense PA according to WHO recommendations, future surveys should be implemented adopting the validated IPAQ-physical activity questionnaire [50]. In this way, it would be possible to provide in-depth knowledge on student PA habits and history. In addition, the data were collected through self-administered questionnaires, which could potentially lead to information bias due to factors such as recall or social desirability. Ecologically sound methods would help assess the veracity of information reported by individuals. Despite the methodological limitations of self-reported measures, they serve as a significant component in comprehending a phenomenon [51] (pp. 224–239), and possess advantages, such as ease of use and cost-effectiveness. Finally, it is important to acknowledge that the sample may not be fully representative of the student academic population, as all participants were volunteers and primarily women. However, it is also relevant to underline that the size of the sample is substantial enough to draw reliable conclusions. 

## 5. Conclusions

This study highlighted the interconnection between PA and healthy diet in the Bicocca student community. A third of the students demonstrate sedentary behavior accompanied by an inadequate nutritional habit. Therefore, it would be beneficial to promote student engagement in cooking and food workshops, as well as nutritional talks and spaces dedicated to PA practice. This approach would transform the universities into healthy hubs, with campuses providing healthier foods at controlled prices. Bicocca is already engaged in changes, moving towards sustainability and promoting well-being consciousness at this stage of student life, according to the goals of the public health agenda: the habits acquired during a university career would be maintained in the years to come. Finally, we must take into account that data collected represent an assessment before the pandemic (COVID-19), thus it would be important to re-run the survey currently after home-confinement and resumption in activity, with a follow up questionnaire after one year, during which events and workshops on these topics will be proposed.

## Figures and Tables

**Table 1:** Descriptive statistics for the adequacy of consumption of foods.

How Often Do You Eat the Following Foods?	% Adequate Consumption
Bread, pasta, rice	81.1
Cured meats	33.3
Chicken, turkey, rabbit, veal	68.0
Beef	43.6
Pork (except cured meats)	64.5
Milk, cheese, dairy products	85.2
Eggs	51.2
Fish	52.4
Cooked and raw leafy vegetables (spinach, salads, chicory, cabbage, broccoli)	47.9
Tomatoes (no preserves), aubergines, peppers, fennel, courgettes, artichokes, carrots, pumpkins, cauliflower, peas, and other fresh legumes	51.9
Fruit	69.2
Dried or canned legumes	39.8
Potatoes	48.6
Salty snacks (chips, popcorn, pretzels, olives)	68.4
Sweets (filled cakes, snacks, ice cream)	38.6
Cooked seasoning	89.2
Saw seasoning	96.9
Salt	73.0

**Table 2:** Descriptive statistics for the two synthesis lifestyle variables.

	Diet	Physical Activity
Mean (SD)	11.03 (2.57)	2.34 (1.93)
Range	1–18	0–9
Skewness (SE)	−0.11 (0.03)	0.59 (0.03)
Kurtosis (SE)	−0.28 (0.06)	−0.04 (0.06)
Behavior’s classification	Non-Adequate = 27.6% Sufficiently Adequate = 68.8% Fully Adequate = 3.6%	Non-Adequate = 34.1% Adequate = 59.0% Intensive = 7.0%

**Table 3:** Percentage of the adequacy of consumption of foods as a function of the adequacy of Physical Activity (PA). The table shows the statistics relating to the foods in which a statistically significant difference was found according to the adequacy of physical activity.

Foods	% Adequate Consumption			χ^2^ (2, *N* = 6947) (*p*-Value)
	Non-Adequate PA	Adequate PA	Intensive PA	
Beef	47.0	42.7	35.7	25.0 (*p* < 0.001)
Pork	66.1	64.4	57.7	12.2 (*p* = 0.002)
Eggs	46.6	52.5	62.7	48.1 (*p* < 0.001)
Fish	45.4	55.4	61.0	76.1 (*p* < 0.001)
Leafy vegetables	40.9	50.7	58.8	82.9 (*p* < 0.001)
Other vegetables	44.5	55.5	57.7	80.2 (*p* < 0.001)
Fruit	60.3	73.2	78.4	137.3 (*p* < 0.001)
Dried/canned legumes	35.0	41.5	48.5	43.1 (*p* < 0.001)
Salty snacks	63.0	71.4	69.7	49.8 (*p* < 0.001)
Sweets	33.8	40.8	42.9	35.8 (*p* < 0.001)
Cooked seasoning	86.5	90.8	89.3	29.8 (*p* < 0.001)
Salt	67.6	75.8	76.5	54.4 (*p* < 0.001)

Note. See Table 1 for a full description of foods.

**Table 4:** Percentage of the adequacy of consumption of foods as a function of gender. The table shows the statistics relating to the foods in which a statistically significant difference was found according to gender.

Foods	% Adequate Consumption		χ^2^ (1, *N* = 6949) (*p*-Value)
	Male	Female	
Bread, pasta, rice	87.7	78.4	81.3 (*p* < 0.001)
Cured meats	23.5	37.3	123 (*p* < 0.001)
Chicken, turkey, rabbit, veal	69.7	67.3	3.9 (*p* < 0.05)
Beef	29.8	49.4	224 (*p* < 0.001)
Pork (except cured meats)	52.0	69.7	197 (*p* < 0.001)
Milk, cheese, dairy products	87.9	84.1	15.9 (*p* < 0.001)
Eggs	59.7	47.6	84.3 (*p* < 0.001)
Leafy vegetables	40.4	51.1	66.0 (*p* < 0.001)
Other vegetables	40.0	56.8	162 (*p* < 0.001)
Fruit	63.5	71.5	43.2 (*p* < 0.001)
Potatoes	56.8	45.2	76.9 (*p* < 0.001)
Salty snacks	63.9	70.3	27.7 (*p* < 0.001)
Salt	67.6	75.3	42.9 (*p* < 0.001)

**Table 5:** Binomial logistic regression analyzing association between physical activity, gender, and adequacy of beef consumption.

Predictor	B	SE	Z	*p*	Odds-Ratio	95% CI	
						Inferior	Superior
Gender (Woman–Man)	0.7107	0.1042	6.818	<0.001	2.035	1.659	2.497
Adequate PA—Non-adequate PA	−0.1849	0.1120	−1.651	0.099	0.831	0.667	1.035
Intensive PA—Non-adequate PA	−0.5032	0.1716	−2.933	0.003	0.605	0.432	0.846
Intensive PA—Adequate PA	−0.3183	0.1567	−2.031	0.042	0.727	0.535	0.989
(Woman–Man) * (Adequate PA—Non-adequate PA)	0.0910	0.1269	0.717	0.473	1.095	0.854	1.405
(Woman–Man) * (Intensive PA—Non-adequate PA)	0.5111	0.2239	2.283	0.022	1.667	1.075	2.585
(Woman–Man) * (Intensive PA—Adequate PA)	0.4200	0.2109	1.991	0.046	1.522	1.007	2.301

Note. The last three rows of the table (with the wording that refers to the levels of the variable Gender * the levels of the variable Physical Activity) express the moderation effect of Gender on the association between Physical Activity and beef consumption. Comparisons between the levels of the two independent variables (Gender and Physical Activity) suggested lower adequacy in beef consumption in respondents who practiced intensive physical activity compared to respondents who practiced insufficient (*p* = 0.022) or adequate physical activity (*p* = 0.046); this difference was characteristic of men and not women (see Figure 1).

## Data Availability

The data presented in this study are available on request from the corresponding author. The data are not publicly available due to privacy and ethical restrictions.

## Supplementary Materials

The following supporting information can be downloaded at: https://www.mdpi.com/article/10.3390/ijerph21050626/s1, Table S1: Percentage of respondents that selected specific responses about the frequency of food consumption; Table S2: Percentage of respondents that selected specific responses about fat consumption; Table S3: Percentage of respondents that selected specific responses about salt consumption; Table S4: Percentage of respondents that selected specific responses about physical activity; Table S5: Types of physical activity done by the sub-sample that overall fall into the intensive physical activity classification (N = 485, 7% of the total sample).
