# Supplementary material for: Health-Related Lifestyles among University Students: Focusing on Eating Habits and Physical Activity
Source: Int J Environ Res Public Health. 2024 May 15;21(5):626. doi: 10.3390/ijerph21050626 (PMC11120768; doi:10.3390/ijerph21050626)
Supplement: Supplementary file 1 [file ijerph-21-00626-s001.zip › Supplementary Material S1.pdf]

Table S1. Percentage of respondents that selected specific responses about the frequency of food consumption

How often do you eat the following foods?	**More than once a day**	**Once a day**	**A few times a week**	**Less than once a week**	**Never**	**National Statistics**
Bread, pasta, rice	**34.8**	**46.3**	16.9	1.7	0.2	77.0 *(At least once a day)*
Cured meats	1.5	9.1	56.1	**25.3**	**8.1**	64.0 *(A few times a week)*
White meats	2.5	11.1	**68.2**	13.8	4.5	83.2 *(A few times a week)*
Beef	0.5	3.6	52.2	**35.5**	**8.2**	65.4 *(A few times a week)*
Pork	0.1	1.6	33.6	**46.9**	**17.7**	52.7 *(A few times a week)*
Milk and dairy products	**14.7**	**29.9**	**40.8**	10.8	3.8	32.8 *(At least once a day)*
Eggs	0.3	2.2	**51.3**	41.5	4.7	68.9 *(A few times a week)*
Fish	0.3	1.9	**52.6**	37.9	7.3	58.4 *(A few times a week)*
Leafy vegetables	**20.2**	**27.7**	36.6	10.9	4.8	44.1 *(At least once a day)*
Other vegetables	**20.2**	**31.7**	39.6	6.3	2.2	44.1 *(At least once a day)*
Fruit	**38.6**	**30.6**	21.5	6.9	2.3	63.4 *(At least once a day)*
Dried/canned legumes	0.5	**2.4**	**37.4**	43.6	16.1	52.0 *(A few times a week)*
Potatoes	0.2	1.7	**48.7**	46.4	3.1	*N.D.*
Salty snacks	0.9	3.8	26.8	**50.2**	**18.3**	43.6 *(A few times a week)*
Sweets	4.3	16.4	40.7	**33.1**	**5.5**	57.8 *(A few times a week)*

Note. Percentages in bold indicate adequate consumption (consistent with guidelines). National data are taken from the public database of the national statistical institute (ISTAT, available at http://dati.istat.it/) and refer to the general Italian population between 18-34 years.
