# Supplementary material for: Health-Related Lifestyles among University Students: Focusing on Eating Habits and Physical Activity
Source: Int J Environ Res Public Health. 2024 May 15;21(5):626. doi: 10.3390/ijerph21050626 (PMC11120768; doi:10.3390/ijerph21050626)
Supplement: Supplementary file 1 [file ijerph-21-00626-s001.zip › Supplementary Material S2.pdf]

Table S2. Percentage of respondents that selected specific responses about fat consumption

	**Olive oil**	**Other vegetable fats and oils (seed oil, margarine)**	**Butter or lard**	**National Statistics (Olive oil and other vegetable fats)**
Which of the following fats do you use most frequently for cooking?	**89.3**	6.3	4.4	96.2
Which of the following fats do you use most frequently for raw seasoning of foods (salads, pasta) or sandwiches?	**97.2**	1.7	1.1	97.2

Note. Percentages in bold indicate adequate behavior (consistent with guidelines). National data are taken from the public database of the national statistical institute (ISTAT, available at http://dati.istat.it/) and refer to the general Italian population between 18-34 years.
