# Supplementary material for: Health-Related Lifestyles among University Students: Focusing on Eating Habits and Physical Activity
Source: Int J Environ Res Public Health. 2024 May 15;21(5):626. doi: 10.3390/ijerph21050626 (PMC11120768; doi:10.3390/ijerph21050626)
Supplement: Supplementary file 1 [file ijerph-21-00626-s001.zip › Supplementary Material S3.pdf]

Table S3. Percentage of respondents that selected specific responses about salt consumption

	**I have never paid attention**	**I have reduced its use over time**	**I have always paid attention**	**National Statistics**
Do you pay attention to the amount of salt and the consumption of salty foods?	27.0	**37.6**	**35.4**	58.7

Note. Percentages in bold indicate adequate behavior (consistent with guidelines). National data are taken from the public database of the national statistical institute (ISTAT, available at http://dati.istat.it/) and refer to the general Italian population between 18-34 years that declare to pay attention to salt consumption.
