# Supplementary material for: Health-Related Lifestyles among University Students: Focusing on Eating Habits and Physical Activity
Source: Int J Environ Res Public Health. 2024 May 15;21(5):626. doi: 10.3390/ijerph21050626 (PMC11120768; doi:10.3390/ijerph21050626)
Supplement: Supplementary file 1 [file ijerph-21-00626-s001.zip › Supplementary Material S4.pdf]

Table S4. Percentage of respondents that selected specific responses about physical activity

Do you practice physical/sporting activities?	**Never**	**1-2 days a week**	**3-4 days a week**	**Five or more days a week**
Physical activity (walking or cycling)	7.7	30.1	30.3	31.8
Physical exercise (gym, swimming pool)	42.7	35.5	18.8	3.0
Sports activity (football, volleyball, tennis)	73.8	16.4	8.4	1.4

Note. National data suggests that 41.5% of the population aged 18-34 play sports continuously. 13.7% practice sports occasionally. 22.3% practice some physical activity. 22.5% do not practice either sport or physical activity. National data are taken from the public database of the national statistical institute (ISTAT, available at http://dati.istat.it/) and refer to the general Italian population.
