# Supplementary material for: Health-Related Lifestyles among University Students: Focusing on Eating Habits and Physical Activity
Source: Int J Environ Res Public Health. 2024 May 15;21(5):626. doi: 10.3390/ijerph21050626 (PMC11120768; doi:10.3390/ijerph21050626)
Supplement: Supplementary file 1 [file ijerph-21-00626-s001.zip › Supplementary Material S5.pdf]

Table S5. Types of physical activity done by the sub-sample that overall fall into the intensive physical activity classification (N = 485, 7% of the total sample)

Do you practice physical/sporting activities?	*N*	*%*
Physical exercise (gym, swimming pool …) at least 3-4 times a week	163	33.6
Sports activity (football, volleyball, tennis …) at least 3-4 times a week	91	18.8
Both physical exercise and sports activity at least 3-4 times a week	231	47.6
